# “Sweetwoods” Lignin as Promising Raw Material to Obtain Micro-Mesoporous Carbon Materials

**DOI:** 10.3390/ma16176024

**Published:** 2023-09-01

**Authors:** Ance Plavniece, Galina Dobele, Dmitrijs Djachkovs, Lilija Jashina, Oskars Bikovens, Aleksandrs Volperts, Aivars Zhurinsh

**Affiliations:** Latvian State Institute of Wood Chemistry, Dzerbenes Street 27, LV-1006 Riga, Latvia; galina.dobele@kki.lv (G.D.);

**Keywords:** biomass, “Sweetwood” lignin, alkali activation, porosity, mesopores

## Abstract

Biorefineries with the significant amounts of lignin as a by-product have a potential to increase business revenues by using this residue to produce high value-added materials. The carbon materials from biomass waste increases the profitability of the production of porous carbon used for sorbents and energy production. The purpose of this research is to study the chemical properties of lignin from “Sweetwoods” biorefinery as well as to characterize lignin carbonizates and activated carbons synthesized from them. This paper describes the effect of carbonization conditions (thermal or hydrothermal) on the properties of activated carbon material. It can be concluded that, depending on the carbonization method, the three-dimensional hierarchical porous structure of activated carbon materials based on “Sweetwoods” lignin, has micro- and mesopores of various sizes and can be used for number of purposes: both for high-quality sorbents, catalysts for electrochemical reduction reactions, providing sufficient space for ion mass transfer in electrodes for energy storage and transfer.

## 1. Introduction

Currently, industrial development has led to a constant increase in demand for energy and sustainable energy resources, so there is an intensive search for a replacement for fossil fuels [1]. The future of overcoming of the crisis of the 21st century largely depends on the successful use of biomass and waste from its processing.

Lignocellulosic biomass is a common source of carbon and is of great interest as a precursor to various carbon materials due to low cost, environmental friendliness and availability. Biomass is renewable and can be a source of clean and affordable energy. These advantages of biomass over fossil fuels promote forestry and lignocellulosic residues management as an alternative carbon sources for chemicals, biofuels, and carbon materials [2,3].

Lignocellulosic biomass is composed of three types of polymers: cellulose, hemicellulose and lignin. Lignin as an integral part of wood, which is usually goes to waste or is burned for energy harvesting, since it is the most difficult component to recycle in any other way. It is produced on a large scale during chemical processing of wood at pulp and paper and hydrolysis industries. On the other hand, lignin is a potential raw material resource for use in many areas of the national economy.

A serious obstacle to assessing the use of lignins are their heterogeneity and structural complexity, insolubility in organic solvents, and low reactivity. Therefore, for a long time, lignin was considered a waste that was usual burned to produce energy. Depolymerization of lignin is an obvious strategy to increase its value, as it provides aromatic chemicals. However, this path turned out to be complicated due to low selectivity and the tendency of lignin fragments to recombine to form condensed carbonized by-products [4].

The abundance of kraft lignin from pulp and paper mills, significant amount of waste of hydrolytic lignin in the production of ethanol from cellulose, as well as the desire to increase the profitability of enterprises, prompted scientists to pay renewed attention to this renewable aromatic heteropolymer. Technological directions have emerged where lignin is considered as a feedstock for the production of valuable products and chemicals with high added value. In recent years, lignin has attracted attention as a precursor for the production of new carbon materials [5,6]. The production of carbon materials with a specific porous structure from biomass waste increases the profitability of the production of porous carbon for energy, which explains the attention paid to this topic [7,8,9]. 

The most large-scale use of technical lignins is the synthesis of carbon materials with a developed porous structure. For these purposes, both physical and chemical (ZnCl_2_, H_3_PO_4_, K_2_CO_3_, KOH, NaOH) activations are used [10,11,12,13]. Compared to physical activation, alkaline thermochemical activation of lignin attracts attention because it allows the process to be carried out at lower temperatures (700–800 °C) and provides high porosity (S_BET_ over 2000 m^2^g^−1^) [14,15]. Nanostructured carbon materials are seen as the most promising to meet the future carbon market demands [16,17].

An important property for interaction with an activator is the functional composition of the precursor, which can be changed using various conditions and methods of carbonization. Using hydrolytic lignin as an example, it was shown that the temperature and rate of preliminary carbonization of the precursor affect the activation process and make it possible to control the average pore size. [18]. Hydrothermal carbonation (HTC) is a new active area of research in the synthesis of carbon-rich materials due to its ability to convert wet biomass into valuable products [19,20]. The pyrolysis of isolated lignins differs significantly from the pyrolysis of wood and other lignocellulosic materials in terms of the yield of the main products [21]. Therefore, it should be taken into account that the conditions of carbonization and pyrolysis, such as temperature, environment and process time for the recovered lignins, must be optimized for each type of feedstock from different types of biomass and recovery processes (for example, kraft, organo-solvent, pyrolytic, etc.) [22,23].

The traditional wood processing industry is mainly driven by the end result, often using by-products to produce low-value solutions, so this are of businesses can be improved even further. Currently, biorefineries are focused on the production of biofuels from raw materials with a high sugar content. In this area, it is promising to unite several European companies to implement the “Sweetwoods” project, which involves the processing of 90% of hardwoods into valuable biomaterials and bioproducts suitable for further use [24]. The new pretreatment technology, combined with the innovative use of fermentation, achieves sugar recovery levels of over 90% and the formation of lignin as a by-product. Biorefineries, which will generate significant amounts of lignin in the future, have the potential to improve business revenues by using lignin to produce high value-added materials [25]. Similar studies are currently being conducted in many scientific centers. Currently, there are not enough studies investigating how the type of lignin affects the resulting hydrothermal structure of the carbonizate.

The purpose of this work is to study the chemical properties of lignin from the “Sweetwoods” biorefinery, as well as to obtain carbonizates from it using both pyrolysis and hydrothermal treatment, with the aim to synthesize activated carbon materials with controlled porosity for use as highly effective sorbents.

## 2. Materials and Methods

### 2.1. Materials and Their Carbonization

The “Sweetwoods” lignin was produced from birch wood by Sunburst^TM^ extrusion based pre-treatment technology [26]. Cellulose was degraded by enzymatic hydrolysis and separated from lignin, which was kindly provided by Fibenol OÜ. Hydrothermal carbonization (HTC) was performed as a pretreatment: 200 g of birch “Sweetwoods” lignin (SW) were dispersed in 2000 mL of distilled water and transferred into a 7.5 L stainless steel PARR-4554 autoclave (Parr Instrument Company, Moline, IL, USA) ). The carbonization process was carried out at 160 (for 4 h), 200(for 4 h), 260 °C (for 2, 4, 6, 8 h), stirring at 140 rpm. To obtain a porous material thermochemical activation was performed at 800 °C using NaOH as an activator (char:activator ratio 1:3), the process is described in detail elsewhere [27].

### 2.2. Analytical Methods Applied for the Materials Study

Carbonized samples were testing using FTIR (Thermo Fisher Nicolet iS50 spectrometer (Thermo Fisher Scientific, Waltham, MA, USA)), Py-GC/MS: Micro Double-shot Pyrolyzer Py-2020iD ((Frontier Laboratories, Koriyama, Japan) directly coupled with Shimadzu GC/MS—QP 2010 apparatus (Shimadzu, Kyoto, Japan) with capillary column RTX-1701 (Restec, Bellefonte, PA, USA)) and TGA using Discovery TGA System (Thermo Fisher Scientific, Waltham, MA, USA), while the methods are described in more detail elsewhere [28]. Carbon, nitrogen, hydrogen and oxygen content was evaluated using the Vario Macro CHNSO (Elementar Analysensysteme GmbH, Langenselbold, Germany) device. 

Porous structure parameters were determined using a Nova 4200e device (Quantachrome, Boynton Beach, FL, USA) using proprietary software (Quantachrome, Boynton Beach, FL, USA) for data analysis for BET (Brunaer-Emmet-Teller) specific surface areas and Density Functional Theory (DFT) for pore size distribution [29]. 

## 3. Results

### 3.1. Chemical Properties of SW Lignin before and after Hydrothermal Carbonization and Pyrolysis

Biomass-based materials differ from fossil coals by the presence of components with different thermal stability and a fairly high content of elemental oxygen. The use of various carbonization methods makes it possible to reduce and, if necessary, control the content of heteroatoms in the structure for the synthesis of micro- or micromesoporous carbon materials using wood and waste from its chemical processing as raw materials [27]. In this study of “Sweetwood” lignin (SW), hydrothermal carbonization (HTC) and pyrolysis were used to produce alkali-activated carbons. It is known that HTC is preferred over the traditional high temperature carbonization method due to low energy consumption and easier control of parameters [30]. The HTC method is also valuable because it provides a higher yield of carbonizate.

Table 1 shows the yields and elemental analysis of SW and samples after pyrolysis and HTC. With an increase in the temperature of HTC treatment from 160 to 260 °C with different duration of the process the yield of carbonizates decreases and the carbon content increases. Under conditions of pyrolysis, the yield of carbonizate decreases even more intensively (Table 1).

**Figure 1:**
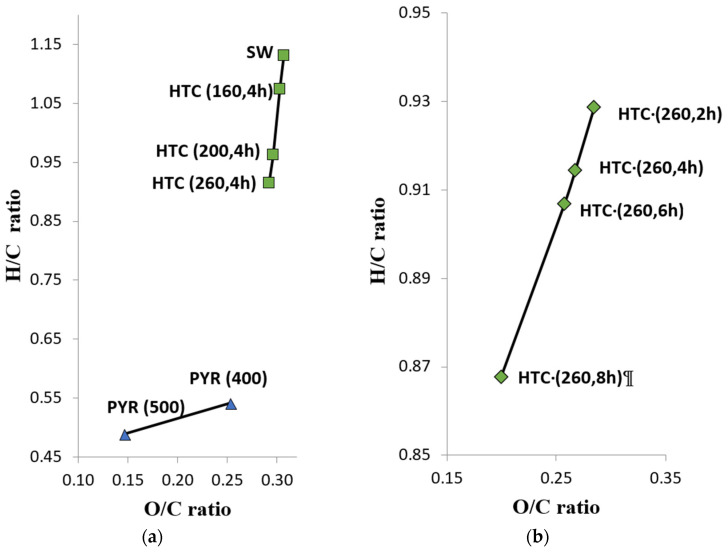
Van Krevelen diagrams for the SW after pyrolysis and hydrothermal carbonization (**a**) at different temperatures, (**b**) 260 °C at different carbonization times.

The decrease in the atomic ratios of oxygen and hydrogen to carbon shows the effect of dehydration and decarboxylation reactions as a result of hydrothermal carbonization at different temperatures and times (Figure 1). These results indicate that increasing the temperature from 160 to 260 °C has more pronounced effect than increasing the reaction time from 2 to 8 h at 260 °C. This coincides with previously published results [31]. In addition, the decrease in H/C and O/C atomic ratios showed a linear relationship with a correlation coefficient of r = 0.999 with increasing reaction time.

**Figure 2:**
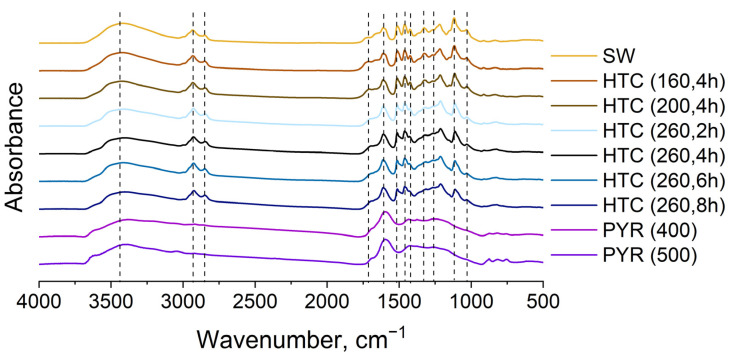
FTIR spectra of SW lignin before and after hydrothermal carbonization (different temperature and at different carbonization time (at 260 °C)), and pyrolysis.

FTIR spectra were recorded to assess the effect of carbonization temperature and time on the composition of functional groups in the structure of carbonized materials. FTIR spectrum of SW-lignin showed typical hardwood lignin (guaiacyl-syringyl type lignin) spectrum with dominant absorption maximum at 1116 cm^−1^ typical for aromatic C-H in plane deformation of lignin syringyl units (Figure 2).

The FTIR absorption peaks were assigned in accordance with [32,33]: 3424 cm^−1^ for O-H stretch, 2929 and 2850 cm^−1^ for C-H stretch in methyl and methylene groups, 1600 and 1514 cm^−1^ for aromatic skeletal vibration, 1463 cm^−1^ for aliphatic C-H deformations, 1428 cm^−1^ for aromatic skeletal vibrations combined with C-H in plane deformations, 1326 cm^−1^ for syringyl and/or condensed guaiacyl units of lignin, 1269 cm^−1^ for guaiacyl units and carbonyl group stretch, 1035 cm^−1^ a complex band for aromatic C-H deformation, C-O-C stretching and C-O-H bending. Regarding methoxy groups, there is currently no general consensus as to where the resonances associated with methoxy groups are located. These resonances are expected to occur at 1265, 1210, 1190, 1125, 1090–1075, 1040 and 1031 cm^−1^ but many of these maximums overlap with other lignin groups [34].

The SW lignin contains unconjugated carbonyl groups (absorption maximum at 1712 cm^−1^). After carbonization carbonyl group stretching vibration absorption maximum was shifted to 1700 cm^−1^ at 160 °C and approximately to 1690 cm^−1^ at 260 °C that revealed carbonyl group conjugation with C=C double band. With increasing of the temperature of the hydrothermal carbonization decreased absorption maximum around 3420 cm^−1^ that indicate decreasing of hydroxyl group content. Relative content of aromatic groups increased (absorption bands at 1600 and 1511 cm^−1^) but C-O-C and C-O-H group vibration intensity decreased (absorption bands at 1123 and 1035 cm^−1^) after the hydrothermal carbonization of the SW at 160 °C. Spectral condensation index, calculated as the dividing between sum of all minima between 1500 and 1050 cm^−1^ and sum of all maxima between 1600 and 1030 cm^−1^ [32], confirms the formation of new C-C-bonds, indicating the formation of lignin condensation, especially at 260 °C. This is also indicated by the decrease in the intensity of the stretching band of the C-O-C group at 1113 cm^−1^ (Figure 2). FTIR results are consistent with other analyses.

To characterize the changes in the SW-lignin under the conditions of hydrothermal treatment, the composition and content of volatile products were studied by analytical pyrolysis and compared to those of the initial lignin (Table 2). The results of the analysis show that the pyrolysis products of the initial lignin contain a small amount (7.26%) of derivatives originating from carbohydrate components. The main part of the aromatic products of pyrolysis are guaiacyl and syringyl derivatives −56.4%. The content of non-methoxylated aromatic compounds is 4.82%. The content of volatile compounds, including carbon dioxide, water and methanol, is 28.2%.

Changes in the structure of lignin begin during hydrothermal treatment already at a temperature of 160 °C and continue to develop with increasing of temperature. First of all, this manifests in the decrease in the content of all products formed from carbohydrates, except for the methyl derivatives of cyclopentane, the content of which almost doubles. The total content of aromatic derivatives of lignin with a phenylpropane structure during hydrothermal treatment at 200 and 260 °C slightly decreases due to increase in the relative proportion of demethoxylated and highly volatile derivatives. It should also be noted that under hydrothermal conditions guaiacil derivatives are less susceptible to changes compared to syringyl derivatives. With an increase in the temperature of hydrothermal treatment from 200 to 260 °C, the trend of such changes increases. However, an increase in the duration of treatment from 4 to 8 h at 260 °C practically does not change the relative content of the products.

Py-GC/MS analysis of pyrolyzed samples showed that the main volatile pyrolysis products belong to the class of benzenes and phenylpropane compounds while methoxy groups characteristic for lignin pyrolysis are absent.

**Figure 3:**
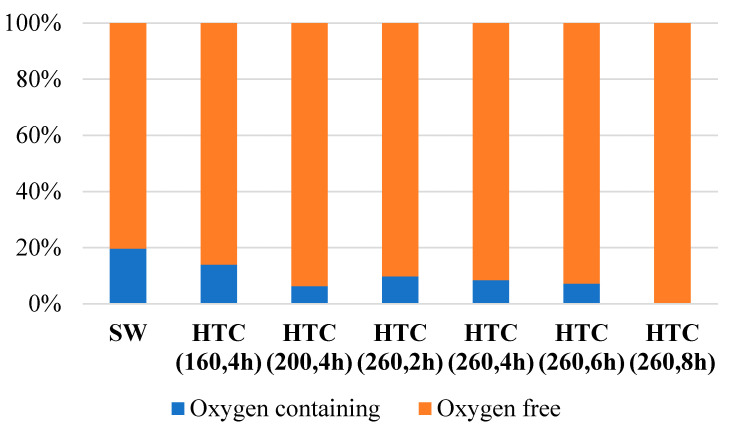
Relative content of volatile pyrolysis products of HTC obtained SW lignin carbonizates containing (blue) and not containing (orange) oxygen in the aliphatic chain (data from Py-GC/MS).

The volatile pyrolysis products of SW lignin, as well as its HTC carbonizates, contain various compounds, including those with functional oxygen-containing groups, belonging to both guaiacol and syringol derivatives (vanillin, acetoguaiacon, propioguaiacone, etc.). The content of compounds with oxygen-containing groups in the aliphatic chain characterizes the degree of change in the lignin macromolecule during carbonization. Figure 3 shows the ratio of the content of volatile pyrolysis compounds with and without oxygen atoms in the propane chain for SW lignin and its HTC carbonizates. The decrease in the content of oxygen-containing compounds in the pyrolysis products is consistent with the FTIR data.

**Figure 4:**
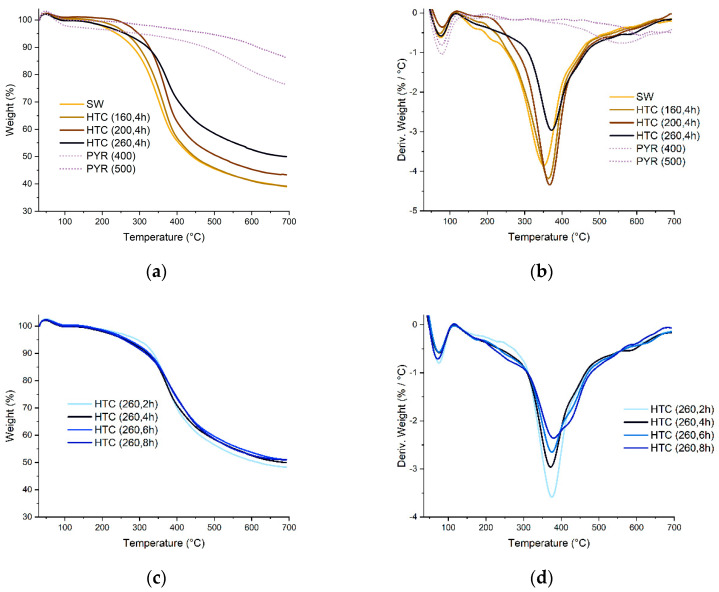
TGA (**a**,**c**) and DTG (**b**,**d**) of SW lignin and its carbonizates ((**a**,**b**) at different carbonization temperatures, (**c**,**d**) HTC at 260 °C carbonization time 2, 4, 6, 8 h).

The TGA results show that the pyrolyzed carbonizates are significantly more thermally stable than those obtained under HTC conditions (Figure 4a,b). Py-GC/M and TGA results, as well as FTIR, indicate the formation of condensed structure during pyrolysis. The thermal stability of the HTC samples increases as the processing temperature rises to 260 °C. An increase in the HTC processing time at 260 °C practically does not change the thermal stability of the samples, only slightly reducing the rate of formation of volatile products (Figure 4c,d).

### 3.2. The Porous Structure of Activated Carbons Depending on the Method of Carbonization

The high specific surface area (BET), controllable porous structure and low cost, of activated carbon materials based on biomass, including chemical processing residues such as lignins, allow them to compete with nanotubes and other carbon materials, the production of which is limited by the complexity and high cost of technologies [29]. Provided that highly efficient porous carbon materials are obtained, the advantage of pyrolysis technologies lies in simple scaling and the ability to control process parameters, depending on the required properties of the final product.

The use of alkali as an activator under the condition of varying process temperature and the amount of the activator, as well as taking into account the properties of precursors and carbonizates, makes it possible to obtain highly porous carbon materials with a developed specific surface due to the formation of large volumes of micropores in the porous structure (Table 3). However, when the same activator is used, the indicators of the porous structure are very different for lignins of different origin. The use of black liquor and Kraft lignin as a precursor for activation leads to the formation of large volumes of mesopores both with and without carbonization of residues [6,35]. However, the use of these precursors, HTC pretreatment, and activation with the alkali such as KOH, are not the only conditions for the formation of a mesoporous structure [36,37]. There is evidence that, upon activation with alkali without preliminary carbonization, a carbon material with a mesopore volume of 78.6% can be obtained from lignin as well [38].

Thermally initiated reactions of the interaction of the alkaline component with the functional groups of lignin proceed simultaneously alongside with the processes of condensation and depolymerization of macromolecules and lead to the development of nanoporous structure of the carbon material.

In this work SW lignin carbonizates were activated with NaOH (activator to precursor ratio 3:1, temperature 800 °C). The yields of activated carbons after HTC and activation was from 5.3 to10.8%, for pyrolyzed and activated samples yield was 14.8 and 15.5% (Table 3).

**Figure 5:**
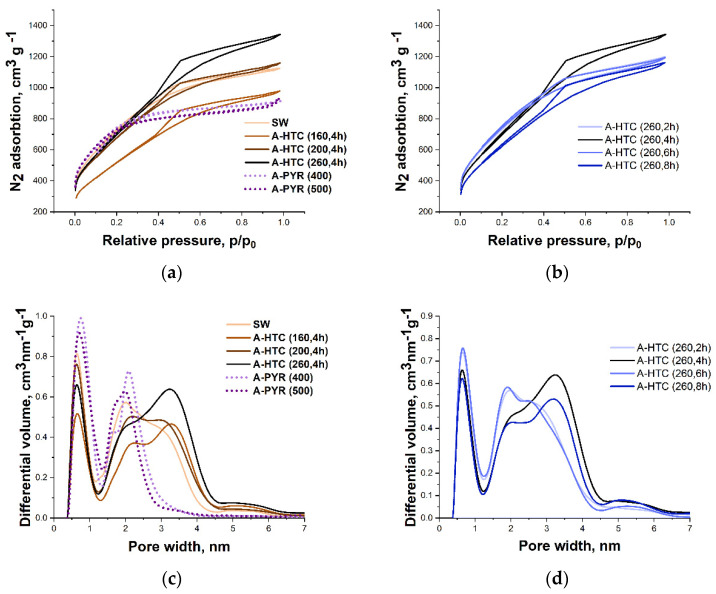
N_2_ adsorption-desorption isotherms (**a**,**b**) and pore size distribution (**c**,**d**) of SW activated carbon depending on temperature (**a**,**c**) depending on carbonation time at 260 °C (**b**,**d**).

According to the shape of nitrogen adsorption–desorption isotherms, all samples of activated carbons obtained both on the basis of HTC and pyrolyzed precursors belong to micro-, mesoporous materials with specific surfaces of more than 2000 m^2^ g^−1^ (Table 4, Figure 5). However, judging by the slope of nitrogen adsorption isotherms, their pore size distributions of samples under study are different. Samples obtained on the basis of thermally carbonized precursors have a pronounced bimodal microporous structures with differential volumes peaks on the pore size distribution curves over pore widths of 1 and 2 nm (Figure 5c,d), and the total volumes in these cases were less than that for HTC samples. For samples of activated carbons based on hydrothermally carbonized precursors and initial lignin, the volume of pores with a width of 1 nm decreases and, as the processing temperature increases, the volumes of mesopores with sizes of 2.5–4.5 nm increase (Table 4, Figure 5c,d).

The calculation of mesopore volumes relative the total volume shows that using various carbonization methods it is possible to increase mesopore volumes from 38–40% for pyrolyzed samples to 52–62% in the case of hydrothermal carbonization.

Thus, it has been demonstrated that the use of various methods of carbonization (thermal and hydrothermal) of SW lignin, followed with NaOH activation, makes it possible to obtain carbon materials with a developed specific surface area of more than 2500 m^2^ g^−1^ with a various pore size distribution. The benefit of using hydrothermal carbonization is a possibility to obtain porous carbons from biomass wastes with a mesopore volume of more than 50%, which is in demand in many sorption processes, as well as catalysts in energy devices (fuel cells, supercapacitor electrodes, ion batteries) [41,42,43].

## 4. Conclusions

The properties of “Sweetwoods” hydrolysis lignin and its carbonizates obtained in the conditions of pyrolysis (400, 500 °C) and hydrothermal treatment (160, 200, 260 °C) were studied by Py-GC/MS, IR-Fourier spectroscopy, and TGA. It is shown that during hydrothermal treatment, oxygen-containing functional groups remain in the structure of carbonized materials, while carbon content increases with the increase of temperature and time of treatment. Pyrolysis leads to intense dehydration and condensation of lignin with the formation of more thermally stable structure.

Activation with NaOH (3:1, 800 °C, 2 h), regardless of the chemical composition and structure of carbonizates, leads to the synthesis of activated carbons with a developed porous area of more than 2000 m^2^ g^−1^ (BET). The influence of carbonization conditions is manifested in the increase in the total pore volume and mesopores widths (2.5–4 nm) after hydrothermal treatment.

It was concluded that using pyrolysis or hydrothermal carbonization it is possible to control micro- and mesopores ratios in the three-dimensional hierarchical porous structure of alkali-activated carbon materials based on “Sweetwoods” lignin. In both cases the obtained materials can be used as high-quality sorbents or catalysts, which is especially true for hydrothermal treatment, since these carbons can provide more space for ion transport in electrodes for energy storage and transfer.

## Figures and Tables

**Table 1:** Elemental analyses and atomic ratios of the SW lignin before and after hydrothermal carbonization and pyrolysis.

Sample	Synthesis Conditions: Temperature and Time	Yield, %	C, %	H, %	O, % *	O/C	H/C
SW	-	-	63.83	6.01	29.54	0.31	1.13
HTC (160, 4 h)	160, 4 h (H_2_O)	88.7	65.68	5.88	27.87	0.30	1.07
HTC (200, 4 h)	200, 4 h (H_2_O)	64.3	67.81	5.43	26.35	0.30	0.96
HTC (260, 2 h)	260, 2 h (H_2_O)	62.0	68.35	5.29	25.94	0.31	0.93
HTC (260, 4 h)	260, 4 h (H_2_O)	59.9	69.55	5.30	24.78	0.29	0.91
HTC (260, 6 h)	260, 6 h (H_2_O)	59.4	70.12	5.30	24.09	0.28	0.91
HTC (260, 8 h)	260, 8 h (H_2_O)	58.8	73.99	5.35	20.07	0.23	0.87
PYR (400)	400, 1 h (pyrolysis)	51.8	80.65	3.64	14.72	0.25	0.54
PYR (500)	500, 1 h (pyrolysis	45.9	87.10	3.55	8.36	0.15	0.49

* O%—oxygen content is calculated as 100% − (H% + C%) = O%, taking into account the content of ash elements.

**Table 2:** Composition of volatile degradation products of SW-lignin and its HTC carbonizates by Py-GC/MS at 500 °C.

	SW	HTC 160	HTC 200	HTC 260			
				2h	4h	6h	8h
**Carbohydrates, %**	**7.26**	**4.23**	**3.38**	**2.06**	**2.49**	**2.50**	**2.36**
Acid, Ester	2.76	1.78	1.45	1.45	1.74	1.75	1.61
Aldehyde, Ketone	2.59	1.21	0.92	-	-	-	-
Cyclopentane derivates	0.29	0.23	0.43	0.39	0.50	0.45	0.52
Furan	1.62	0.94	0.58	0.22	0.25	0.30	0.23
Pyran	trace	0.07	-	-	-	-	-
**Lignin derivates, %**	**61.21**	**61.49**	**59.32**	**59.28**	**56.34**	**56.46**	**56.83**
Phenyl and benzyl derivates	4.82	4.92	6.07	6.96	8.98	8.99	8.05
Guaiacyl derivates	17.69	17.93	16.97	16.01	15.78	16.22	16.09
Syringyl derivates	38.70	38.64	36.28	36.31	31.58	31.25	32.69
**Summary: Carbon dioxide, Water, Methanol, %**	**28.21**	**31.06**	**32.53**	**33.97**	**35.72**	**35.38**	**35.80**
**N-containing, %**	0.68	0.47	0.40	0.02	0.03	0.03	0.02
**Other compounds, %**	1.61	1.60	3.08	3.40	4.35	4.84	4.09
**Identified, %**	**98.97**	**98.85**	**98.71**	**98.73**	**98.93**	**99.21**	**99.10**

**Table 3:** Lignin bases activated carbon porous structure comparison.

Lignin	Carbonization Conditions: Temperature, Environment	Activation Conditions: Temperature, Activator (Activator to Carbon Material)	Porous Structure			Ref.
			S_BET_, m^2^g^−1^	V_t_, cm^3^ g^−1^	V_meso_ from Vt, %	
Black liquor		800 °C NaOH (2:1)	* 2104	* 2.35	* 71	[6]
Lignin (Kraft lignin, USA)	180 °C HTC (acidic)	800 °C KOH (1:1)	1374	0.75	72	[35]
Lignin (Tokyo Chemical Industry Co., Ltd., Tokyo, Japan)	100 °C 150 °C 200 °C 250 °C HTC (H_2_O)	900 °C KOH (2:1)	2448 3033 3178 2694	1.32 1.93 1.94 1.55	17.42 34.72 29.90 24.52	[36]
Precipitated lignin		800 °C KOH	* 1043	* 0.58	20.69	[37]
Dealkalized lignin	350 °C	800 °C 600 °C KOH (2:1)	2050	0.84	9.52	[39]
			1659	0.71	11.27	
	450 °C					
			1493	0.60	10	
	550 °C					
			1407	0.57	8.77	
Lignin (chemical plant in Shandong province, China)	- 600°C	800 °C KOH (3:1)	* 2889 2233	* 1.44 1.06	78.57 28.30	[40]
Larch Klason lignin Organosolv lignin	600°C	900 °C	2451 2304 2388	1.01 0.94 0.97	5.9 5.3 6.2	[38]

* Without carbonization.

**Table 4:** Yields, porous structure parameters and elemental composition of SW activated carbon.

Sample	Yield (After Carbonization and Activation), %	Specific Surface Area (BET), m^2^ g^−1^	Pore Volume, cm^3^ g^−1^			Average Pore Width, nm	Mesopores from Vt, %	N, %	C, %	H, %	O, %
			Total	Micro	Meso						
A-SW	5	2655	1.7	0.8	0.9	2.6	52.8	0.72	93.98	1.61	3.69
A-HTC (160, 4 h)	5.3	2000	1.5	0.6	0.9	3.2	60.0	0.89	85.49	0.70	12.92
A-HTC (200, 4 h)	6.9	2485	1.8	0.8	1.0	2.9	56.6	0.66	90.16	1.31	7.87
A-HTC (260, 2 h)	9.1	2778	1.9	0.9	1.0	2.7	53.8	0.47	92.85	0.30	6.38
A-HTC (260, 4 h)	10.5	2555	2.1	0.8	1.3	3.3	61.5	1.29	92.23	0.38	6.11
A-HTC (260, 6 h)	10.8	2727	1.8	0.8	1.0	2.7	54.3	1.30	92.20	0.34	6.16
A-HTC (260, 8 h)	11.5	2286	1.8	0.7	1.1	3.1	60.4	1.19	89.48	0.82	8.52
A-PYR (400)	14.8	2729	1.4	0.9	0.5	2.1	38.0	0.58	91.82	0.26	7.34
A-PYR (500)	15.5	2674	1.4	0.9	0.6	2.2	39.5	0.73	94.53	0.28	4.46

## Data Availability

The data presented in this study are available on request from the corresponding author.
